# Association between serum aluminum levels and cardiothoracic ratio in patients on chronic hemodialysis

**DOI:** 10.1371/journal.pone.0190008

**Published:** 2017-12-20

**Authors:** Tzu-Lin Wang, Yu-Wei Fang, Jyh-Gang Leu, Ming-Hsien Tsai

**Affiliations:** 1 Division of Cardiology, Department of Internal Medicine, Shin-Kong Wu Ho-Su Memorial Hospital, Taipei, Taiwan, R.O.C.; 2 Division of Nephrology, Department of Internal Medicine, Shin-Kong Wu Ho-Su Memorial Hospital, Taipei, Taiwan, R.O.C.; 3 Fu-Jen Catholic University School of Medicine, Taipei, Taiwan, R.O.C.; 4 Division of Biostatistics, Institutes of Epidemiology and Preventive Medicine, College of Public Health, National Taiwan University, Taipei, Taiwan, R.O.C.; Kurume University School of Medicine, JAPAN

## Abstract

The cardiothoracic ratio (CTR) and serum aluminum levels are both associated with mortality in hemodialysis patients. However, limited data regarding the association between serum aluminum levels and the CTR have been published to date. Therefore, we aimed to elucidate this association in patients on chronic hemodialysis (CHD). We investigated the association between the serum aluminum level and the CTR in CHD in a retrospective cross-sectional study of 547 Taiwanese patients on CHD. The mean age of patients was 62.5±13.2 years, with a mean hemodialysis time of 7.1±5.2 years. Among the patients, 36.9% were diabetic and 47.9% were male. After natural logarithmic transformation (ln(aluminum)), the serum aluminum level exhibited an independent and linear relationship with the CTR (β: 1.40, 95% confidence interval (CI), 0.6–2.2). A high serum aluminum level (≥6 ng/dL) was significantly associated with a CTR >0.5 in the crude analysis (odds ratio (OR): 2.15, 95% CI, 1.52–3.04) and remained significant after multivariable adjustment (OR: 2.45, 95% CI, 1.63–3.67). Moreover, the ln(aluminum) value was significantly associated with a CTR >0.5 (OR: 1.71, 95%CI, 1.28–2.29) in multivariable analysis, indicating a dose effect of aluminum on cardiomegaly. In conclusion, the serum aluminum level was independently associated with cardiac remodeling (elevated CTR) in patients on CHD.

## Introduction

The cardiothoracic ratio (CTR) is estimated by measuring the proportion of the heart size to the thoracic diameter on chest radiographs and it has been shown weakly and negatively associated with cardiac systolic dysfunction [1, 2]. Moreover, a CTR >50% is considered cardiomegaly, and an increased CTR will lead to a poor clinical prognosis in the elderly [3], patients with heart failure [4–6], hypertension [7] and chronic dialysis [8–14]. Ventricular remodeling refers to changes in the size, shape, structure, and function of the heart [15]. Therefore, the CTR could be representative of the ventricular remodeling status and is a simple method to assess the heart conditions of patients on chronic dialysis.

An elevated aluminum level contributes to the development of dialysis dementia, adynamic bone disease, and anemia in patients on chronic hemodialysis (CHD) [16–18]. Currently, overt aluminum toxicity is uncommon in patients on CHD [19, 20] because the aluminum in the water used for dialysis is removed by reverse osmosis and deionization. However, serum aluminum remains an important issue for patients on CHD due to inefficient removal of aluminum by dialysis and more frequent exposure to aluminum-containing medications [21, 22]. Some studies have reported that elevated serum aluminum levels are associated with mortality in patients on CHD [23, 24]. Moreover, aluminum might have a damaging effect on cardiac remodeling, as evidenced by studies showing a significant association between heart damage and aluminum levels [25, 26].

However, no study has examined the association between the CTR and serum aluminum level in dialysis patients. Therefore, we conducted the present study to investigate their association in patients on CHD.

## Materials and methods

### Study design and patients

We conducted a retrospective cross-sectional study in a single medical center of Shin-Kong Wu Ho-Su Memorial Hospital. The inclusion criteria were that patients had undergone regular hemodialysis for at least three months before being enrolled in the study and must have been clinically stable for three months preceding the study, without hospitalization for any reason. Moreover, patients without serum aluminum level and CTR data were excluded. Thereafter, a total of 547 patients receiving regular hemodialysis in the dialysis unit of the Shin Kong Wu Ho-Su Memorial Hospital were included in the study from December 2009 to December 2012. Just first dataset of every patient was put into analysis. This study was performed in accordance with the principles of the Declaration of Helsinki and was approved by the Ethics Committee of the Shin-Kong Wu Ho-Su Memorial Hospital. Moreover, Informed consent was waived by the Ethics Committee of the Shin-Kong Wu Ho-Su Memorial Hospital because our study was based on a medical chart review. Patient information was anonymized and de-identified prior to analysis.

### Medical and laboratory data

We obtained the data from patients’ medical records, including demographic data and comorbidity and dialysis-related biochemistry results. The information included age, gender, smoking history (never *versus* ever), blood pressure, which was recorded as the mean value of two measurements before the last dialysis session of a week, history of diabetes mellitus (DM), cardiovascular disease (CVD) diagnosed according to a documented history of coronary artery or cerebrovascular disease, body mass index, HD vintage, CTR, serum aluminum and albumin levels, total cholesterol, triglyceride, iron profile, hemoglobin levels, intact parathyroid hormone (iPTH), ionized calcium, phosphate levels, and urea kinetics (Kt/V). Blood samples were collected for laboratory testing after at least 8 h of fasting and before the dialysis session. Biochemical measurements were performed with standard commercial assays and automated testing instruments (Beckman, Lane Cove, NSW, Australia). iPTH was measured using the Roche Elecsys assay (Roche Diagnostics, Basel, Switzerland). Additionally, aluminum was measured by graphite furnace atomic absorption spectrometry using a GBC 906AA (Braeside VIC, Australia).

### Cardiothoracic ratio measurement

Posterior-anterior chest radiographs were obtained to regularly measure the CTR at the end of the year for patients on CHD after a mid-week HD session at our medical center. We used computer software (UniWeb Viewer, EBM Technologies Inc., Taiwan) to ensure the accuracy of measurements. The CTR was calculated by dividing the maximal horizontal width of the heart by the horizontal diameter of inner borders of the rib cage. Therefore, a CTR >0.5 was defined as cardiomegaly, with higher CTR levels indicating a greater severity of cardiomegaly.

### Statistical analyses

Data are expressed as the mean ± standard deviation (SD) or median with interquartile range (25^th^–75^th^ percentile, IQR) as appropriate for continuous variables and as proportions for categorical data. A natural log transformation (ln) was used to approximate a normal distribution if the variable did not have a normal distribution. The Pearson correlation coefficient was adopted to examine the correlations between variables. Linear regression analyses were performed using the CTR as the dependent variable to investigate its association with the ln(aluminum) value. Moreover, a generalized linear model was used to determine the risk of a CTR >0.5 using the link function of logit. Subgroup analysis was also performed and was stratified by the factors of gender (male and female), age (≤60 and >60 years), previous CVD (yes and no), DM (yes and no), and hemoglobin level (≤9 and >9 g/dL) separately. A two-tailed *P*-value of <0.05 was considered statistically significant. All statistical analyses were performed using SAS for Windows version 9.4 (SAS Institute Inc., Cary, NC, USA).

## Results

**Fig 1:**
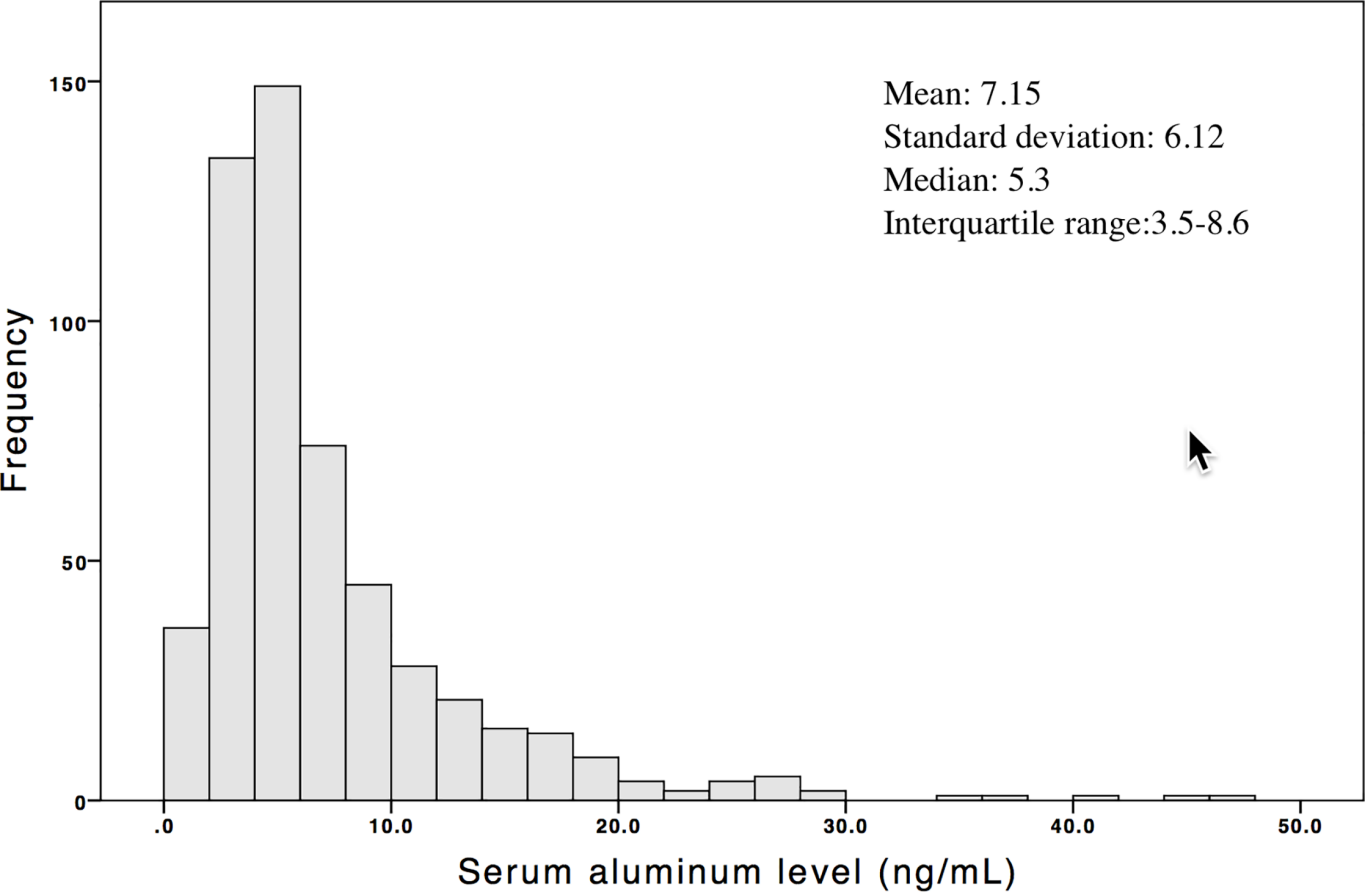
Distribution of serum aluminum levels in patients on chronic hemodialysis.

**Fig 2:**
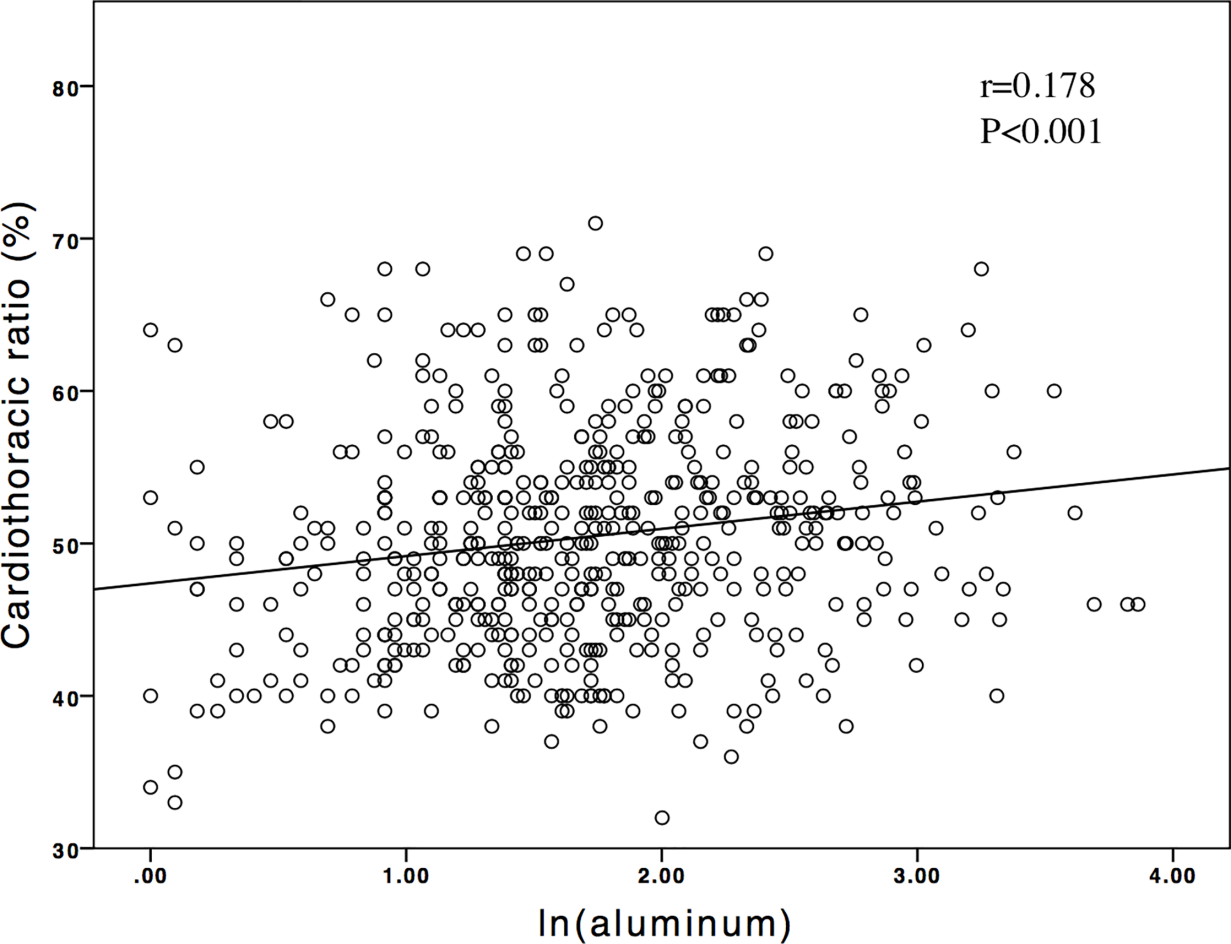
Regression plot of cardiothoracic ratios (%) with serum aluminum levels after natural log transformation (ln(aluminum)). Lines indicate best-fit regression lines derived from the least mean square method.

**Fig 3:**
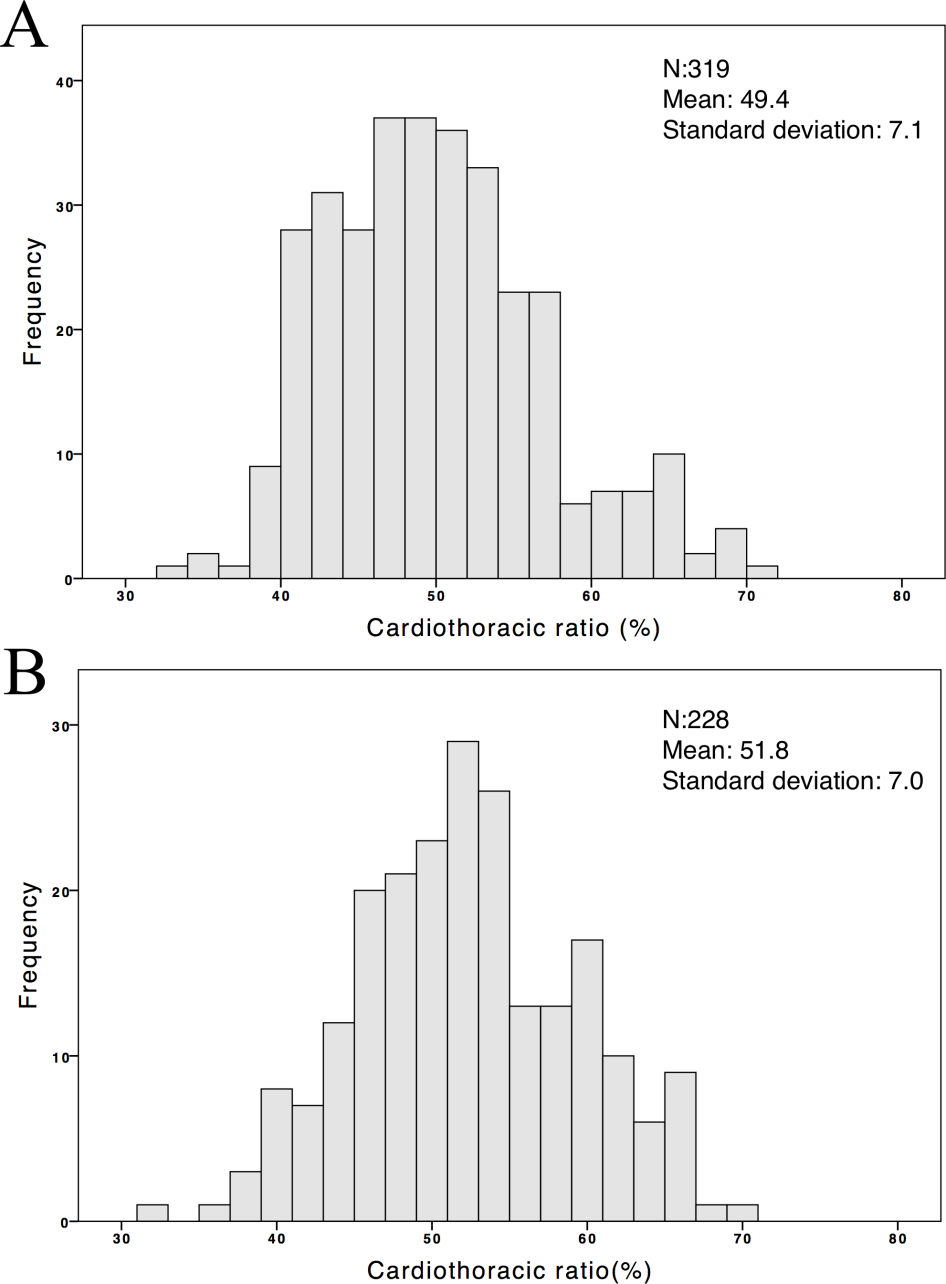
**Distribution of the cardiothoracic ratio (%) in groups of patients on chronic hemodialysis with serum aluminum levels (a) <6 ng/mL and (b) ≥6 ng/mL**.

The mean age of the 547 patients on CHD was 62.5±13.2 years, with a mean HD time of 7.1±5.2 years. Among them, 36.9% were diabetic and 47.9% were male. Two hundred fifty-four (46.4%) patients had a CTR >0.5, and 55 (10%) patients had a CTR >0.6. The mean CTR was 50.4±7.1% (IQR, 46–56%). Moreover, two hundred twenty-eight (41.6%) patients had an aluminum level ≥6 ng/mL and 22 (4%) patients had the level more than 20 ng/mL. The other clinical characteristics of the participants are shown in Table 1. The median serum aluminum level was 5.3 (IQR, 3.5–8.6) ng/mL with a distribution skewed toward the right (Fig 1). Moreover, the significant linear association between ln(aluminum) values and the CTR is shown in Fig 2. The CTR distribution was stratified based on a serum aluminum level of 6 ng/mL, and the group with an aluminum level ≥6 ng/mL had a greater mean CTR (Fig 3).

**Table 1:** Characteristics of the study population.

Characteristics	Values (N = 547)
Age (years ± SD)	62.5±13.2
Males [n (%)]	262 (47.9)
Duration of dialysis (years ± SD)	7.1±5.2
Diabetes mellitus [n (%)]	202 (36.9)
Previous CVD [n (%)]	136 (24.9)
Smoking [n (%)]	108 (20)
Systolic BP (mmHg ± SD)	147±35
Diastolic BP (mmHg ± SD)	77±17
Body mass index (kg/m^2^ ± SD)	23.3±4
Albumin level (g/dL ± SD)	4.1±0.3
Triglyceride (mg/dL ± SD)	122 (81, 187)
Cholesterol level (mg/dL ± SD)	175±42
Kt/V (±SD)	1.6±0.2
Hemoglobin (g/dL ± SD)	10.4±1.3
iPTH (pg/mL ± SD)	126 (55, 268)
Transferrin saturation (% ± SD)	35±14
Ionized calcium (mg/dL ± SD)	4.7±0.4
Phosphate (mg/dL ± SD)	5.1±1.3
SAL (ng/mL, IRQ)	5.3 (3.5, 8.6)
SAL ≥6 ng/mL [n (%)]	228 (41.6)
SAL ≥20 ng/mL [n (%)]	22 (4)
CTR (% ± SD)	50.4±7.1
CTR >0.5 [n (%)]	254 (46)
CTR >0.6 [n (%)]	55 (10)

Abbreviations: SD, standard deviation; CVD, cardiovascular disease; BP, blood pressure; Kt/V, urea kinetics; iPTH, intact parathyroid hormone; SAL, serum aluminum level; IRQ, interquartile range; CTR, cardiothoracic ratio.

### Determinants of the CTR in patients on CHD

Table 2 shows that age, gender, previous CVD, smoking, diastolic BP, ln(aluminum), albumin, hemoglobin, transferrin saturation, ionized calcium, and phosphate were significantly associated with the CTR in the crude analysis. However, after adjusting for multiple variables, only age, body mass index, ln(aluminum), hemoglobin, and transferrin saturation were significantly associated with the CTR.

**Table 2:** Determinants of the cardiothoracic ratio.

	Crude		Multivariable	
Parameter	β (95% CI)	*P*	β (95% CI)	*P*
Age (per year)	0.18 (0.14, 0.23)	<0.001	0.18 (0.13, 0.23)	<0.001
Male versus female	-2.53 (-3.72, -1.35)	<0.001	-0.51 (-1.81, 0.78)	0.435
Duration of dialysis (per year)	0.07 (-0.04, 0.19)	0.198	0.11 (-0.004, 0.23)	0.059
Diabetes mellitus (yes vs no)	1.22 (-0.02, 2.46)	0.054	1.24 (-0.04, 2.53)	0.058
Previous CVD (yes vs no)	2.22 (0.83, 3.60)	0.002	0.69 (-0.61, 1.99)	0.298
Smoking (ever versus never)	-2.37 (-3.87, -0.87)	0.002	-1.33 (-2.83, 0.16)	0.082
Systolic BP (per 10 mmHg)	-0.01 (-0.18, 0.16)	0.887	0.17 (-0.08, 0.43)	0.185
Diastolic BP (per 10 mmHg)	-0.38 (-0.72, -0.04)	0.031	0.12 (-0.43, 0.68)	0.661
Body mass index (per 1 kg/m^2^)	0.09 (-0.05, 0.24)	0.207	0.17 (0.03, 0.31)	0.017
ln(aluminum) (per 1 unit)	1.78 (0.95, 2.61)	<0.001	1.40 (0.60, 2.20)	0.001
Albumin level (per 1 g/dL)	-3.29 (-4.79, -1.79)	<0.001	0.10 (-1.50, 1.70)	0.900
ln(Triglyceride) (per 1 unit)	-0.50 (-1.48, 0.46)	0.307		
Cholesterol level (per 1 mg/dL)	0.001(-0.01, 0.01)	0.962		
Kt/V (per 1 unit)	1.08 (-1.25, 3.43)	0.363		
Hemoglobin (per 1 g/dL)	-0.98 (-1.41, -0.54)	<0.001	-0.65 (-1.09, -0.22)	0.003
ln(iPTH) (per 1 unit)	0.26 (-0.29, 0.82)	0.357		
Transferrin saturation (per 1%)	-0.09 (-0.13, -0.04)	<0.001	-0.07 (-0.11, -0.03)	<0.001
Ionized calcium (per 1 mg/dL)	1.74 (0.53, 2.95)	0.005	0.93 (-0.22, 2.08)	0.113
Phosphate (per 1 mg/dL)	-0.48 (-0.92, -0.05)	0.029	-0.14 (-0.55, 0.26)	0.480

Abbreviation: aOR, adjusted odds ratio; CVD, cardiovascular disease; BP, blood pressure; Kt/V, urea kinetics; iPTH, intact parathyroid hormone

### Association between serum aluminum levels and the CTR

In further stepwise modeling and analysis (Table 3), a higher serum aluminum level (≥6 ng/mL) was significantly associated with a CTR >0.5 (odds ratio [OR], 2.15; 95% confidence interval [CI], 1.52–3.04) in the crude analysis. After further adjustment to demographic variables and comorbidity and dialysis-related parameters, significance was maintained with an OR of 2.45 (95% CI 1.63–3.67). Moreover, the ln(aluminum) value was also significantly associated with a CTR >0.5 (OR, 1.67; 95% CI, 1.30–2.14) in the crude analysis. After further adjustment for confounding variables, significance was maintained with an OR of 1.71 (95% CI 1.28–2.29).

**Table 3:** Logistic regression analysis of risk factors for cardiomegaly (CTR >0.5) in patients on chronic hemodialysis.

	Aluminum cut-off value of 6 ng/mL		Each increment of ln(aluminum)	
	OR (95% CI)	*P* value	OR (95% CI)	*P* value
Crude	2.15 (1.52–3.04)	<0.001	1.67 (1.30–2.14)	<0.001
Model 1	2.36 (1.62–3.45)	<0.001	1.73 (1.32–2.27)	<0.001
Model 2	2.21 (1.50–3.26)	<0.001	1.69 (1.28–2.24)	<0.001
Model 3	2.45 (1.63–3.67)	<0.001	1.71 (1.28–2.29)	<0.001

Multivariate model 1 is adjusted for age, gender, and hemodialysis vintage. Multivariate model 2 comprises model 1 as well as adjustments for diabetes mellitus, cardiovascular disease, smoking, systolic blood pressure, diastolic blood pressure and body mass index. Multivariate model 3 comprises model 2 as well as adjustments for albumin, hemoglobin, transferrin saturation, ionized calcium, and phosphate.

Abbreviation: OR, odds ratio; CI, confidence interval; ln, natural log transformation.

### Subgroup analysis

**Fig 4:**
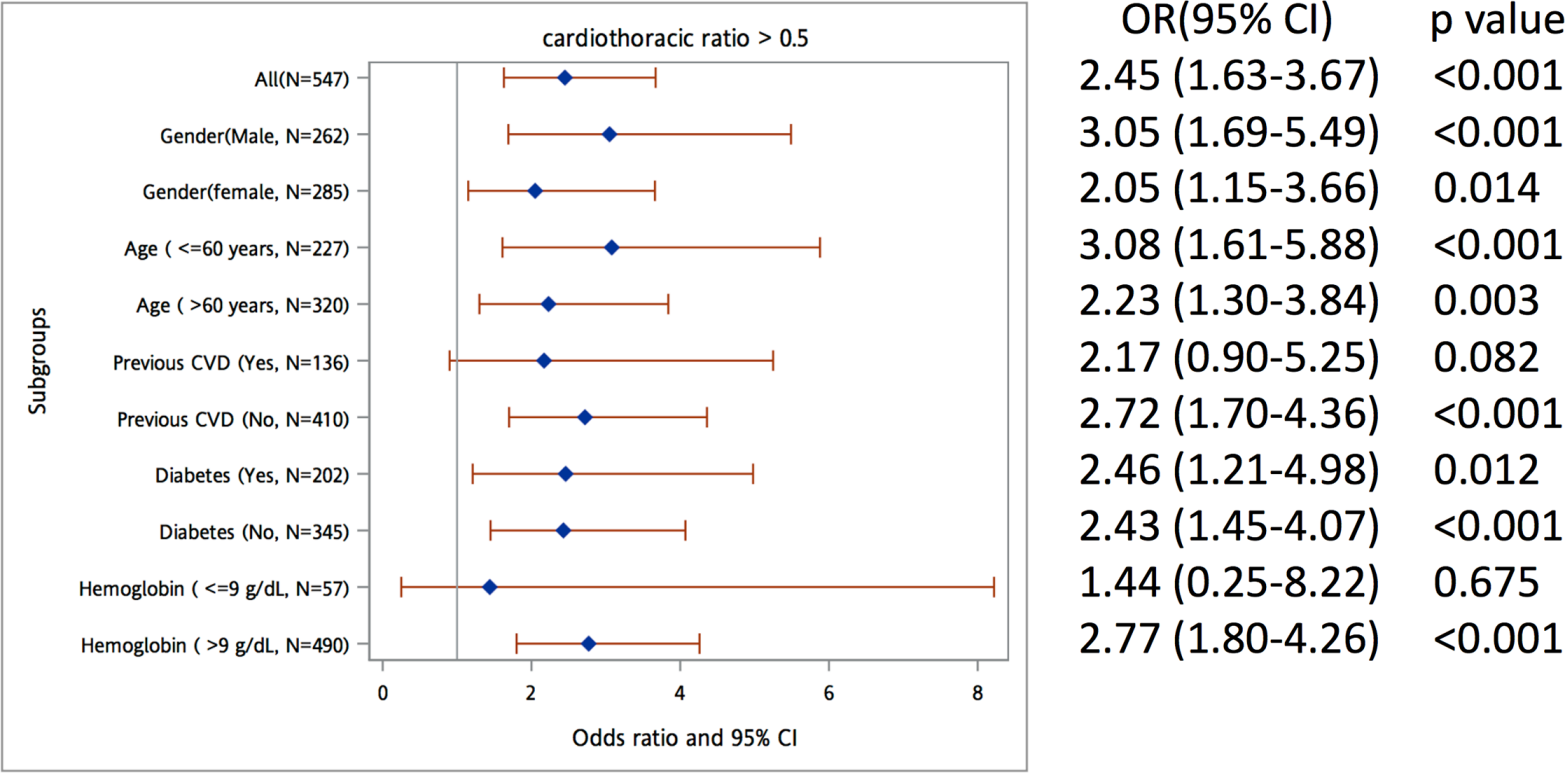
**Subgroup analysis of the association between a cardiothoracic ratio >0.5 and (a) the serum aluminum level with a cut-off value of 6 ng/dL; and (b) the serum aluminum level after natural log transformation (ln(aluminum))**. The full model comprised adjusted variables, including age, gender, hemodialysis vintage, diabetes mellitus, cardiovascular disease, smoking history, systolic blood pressure, diastolic blood pressure, body mass index, albumin, hemoglobin, transferrin saturation, ionized calcium, and phosphate levels.

We investigated the association between the bivariate aluminum level (with a cutoff value of 6 ng/mL) and a CTR >0.5 in analyses stratified by covariates including a history of DM, previous CVD, hemoglobin, age, and gender. Fig 4 shows that a higher aluminum level (≥6 ng/dL) was significantly associated with a CTR >0.5 in the group without CVD and the group with a hemoglobin value >9 g/dL after multivariable adjustment for demographic characteristics and laboratory data regarding HD. The serum aluminum level seems to have no discrepant effect in groups with different genders, ages or DM histories.

## Discussion

In this cross-sectional study of 547 patients on CHD, both higher serum aluminum (≥6 ng/mL) and ln(aluminum) levels were independently correlated with a CTR >0.5. Moreover, a linear association was also noted between ln(aluminum) and continuous CTR values. These relationships were independent of traditional anemia risk factors. This finding emphasizes that aluminum may have a toxic effect on cardiac remodeling in patients on CHD and may serve as an initiator of cardiomegaly. Physicians should maintain serum aluminum levels as low as possible in dialysis patients.

The echocardiography is the optimal method for determination of cardiomegaly and especially left ventricular function because CTR can be increased due to epicardial fat pad, expiration, and fluid overload [27, 28]. However, an autopsy study has reported a satisfactory association between CTR and heart size [29]. Moreover, some studies have disclosed that CTR had significant association with CV mortality [9, 12, 14] or CV events [11, 13] in patients on CHD. Thereafter, an elevated CTR may indicate heart problem in certain extent and then it allows us to use CTR as the surrogate of ventricular remodeling in patients on CHD because CTR is a simple and inexpensive method.

We proposed a hypothesis regarding our finding that the serum aluminum level is independently and significantly associated with cardiomegaly (CTR >0.5) in patients on CHD. A previous in vitro study indicated that aluminum inhibits the regeneration of reduced glutathione, thereby leading to oxidative damage [30]. Oxidative stress induced by aluminum will cause a disturbance of the intracellular redox system and will consequently contribute to cardiomyopathy and atherosclerosis [31], which could partially explain the poor clinical outcomes in patients on CHD. However, the actual mechanism still requires further exploration. Moreover, in our subgroup analysis, a higher serum aluminum level was not significantly associated with cardiomegaly in groups with hemoglobin levels ≤9 g/dL or CVD history. This may have occurred because anemia and CVD are both strong risk factors for the development of cardiomegaly in patients on CHD [32, 33] and may negate the impact of the hazardous effect of aluminum on the heart.

The National Kidney Foundation–Kidney Disease Outcomes Quality Initiative (KDOQI) guideline [34] recommends that physicians should take actions to decrease the serum aluminum level in patients on CHD when it exceeds 20 ng/mL. However, according to our study, we suggest that an early alert is needed, even when the aluminum level is only slightly above the normal range (0–6 ng/mL) due to its hazardous effect on cardiac remodeling. Clinically, dialysis patients have a higher chance of being exposed to aluminum-containing products, including aluminum-containing phosphate binders and antacids, iron and calcium-containing medications, calcitriol, vitamin B complex, erythropoietin, and insulin [21, 35–37]. Aluminum-containing phosphate binders are one of the most common sources of aluminum [38], and the KDOQI has suggested discontinuing their use. However, aluminum-containing phosphate binders are still being used in some countries due to financial reasons or uncontrolled hyperphosphatemia [39]. Therefore, more intensive screening of the serum aluminum level is needed for such patients, and an early response is necessary if the serum aluminum level is beyond the normal range.

Our study had some limitations that should be considered when elaborating the results. First, due to the cross-sectional design, the causal relationship between serum aluminum levels and CTRs cannot be inferred. However, according to previous studies [25, 26] and based on the pathological view, we suggest that an elevated serum aluminum level occurs before cardiomegaly. Second, this was a single-center study, and the results might not be applicable to all CHD populations. Generalization of the results should be clarified in further studies. However, the strong association between the serum aluminum level and CTR >0.5 in our study may extenuate this limitation. Third, some patients might not follow the guideline to take the picture of chest X-ray, which would introduce an overestimate of CTR due to fluid overload. However, this occurred at a random pattern and then the inferred result would not be altered because this bias contributed equally to the groups. Finally, we did not assess the internal variability of the CTR measurement. However, the use of computer-assisted CTR measurements may diminish this bias because this method has been shown to be a simple and reliable method to assess the heart [40].

## Conclusion

A significant and independent association was observed between higher serum aluminum levels (higher than the normal range) and cardiomegaly (CTR >0.5) in patients on CHD. Therefore, exposure to aluminum-containing medication should be avoided as much as possible to impede the elevation of serum aluminum levels in patients on CHD.

## Supporting information

S1 Dataset The collected data in the study. (XLSX)
